# Media and microcarrier surface must be optimized when transitioning mesenchymal stem/stromal cell expansion to stirred tank bioreactors

**DOI:** 10.1186/1753-6561-9-S9-P57

**Published:** 2015-12-14

**Authors:** Aletta Schnitzler, Anjali Verma, Manjula Aysola, Julie Murrell, Martha Rook

**Affiliations:** 1 EMD Millipore Corporation, Bedford, MA, 01730, USA

## Background

The long-term outlook for regenerative medicine predicts an increased need for high quality materials that are compatible with the limited number of downstream processing steps required for cell-based therapies. Large scale manufacturing of adherent-dependent cell types necessitates movement away from planar culture and toward technologies such as stirred tank bioreactors where suspension culture using microcarriers is enabled [1]. Microcarriers are available in a variety of base materials including glass, polystyrene or dextran, and have been coated or derivatized to carry charge, peptides or extracellular matrix proteins such as collagen that may be animal-derived. Cell culture medium may also contain animal-derived components. Fetal bovine serum (FBS) in particular is associated with regulatory, supply, and consistency challenges [2]. Eliminating this commonly-used reagent will require thorough evaluation of animal origin-free materials for compatibility with cell therapy applications. Here, we evaluated growth of human mesenchymal stem/stromal cells (MSCs) with a variety of microcarriers and cell culture media formulations. Not only was a wide range of performance observed between the microcarriers and media screened, but positive performance in static culture was not necessarily predictive of that under agitated conditions.

## Materials and Methods

To evaluate different microcarriers, human adipose-derived MSCs (aMSCs) were grown on a panel of microcarriers in Petri dishes, 125 mL spinner flasks, or Mobius® 3 L bioreactors in DMEM supplemented with 10% FBS and 8 ng/mL bFGF (DMEM FBS). TrypLE was used to detach a MSCs from microcarriers to assess recovery.

To evaluate different media formulations, human bone marrow-derived MSCs (bmMSCs) were grown on gelatin-coated T-flasks for planar culture or seeded onto collagen-coated microcarriers in 125 mL spinner flasks for suspension culture. Planar cultures were maintained in respective media from passage 4 to 8. Top performing media were further evaluated in microcarrier-based suspension culture for 7 days. Serum (DMEMFBS) control flasks were run in parallel.

Cell were seeded at 3k cells / cm2 and 5.4 cm2 / mL of culture surface was provided in all microcarrier experiments. Nexcelom Cellometer® and NucleoCounter® devices were used for cell counting.

## Results

Six commercially-available microcarriers were tested for their ability to support growth, detachment and viability of a MSCs expanded in DMEM FBS. The evaluation was conducted in both static culture (Petri dishes) and agitated culture (spinner flasks). Microcarriers were assigned a rank based on their overall performance in the two platforms (Table 1). Microcarrier C was ranked the best, primarily driven by high cell yields. Interestingly, microcarriers B, D and E supported robust growth in static culture with high day 7 fold increases, but did not perform as well under agitated conditions. Microcarriers A, B and C were further evaluated in the Mobius® 3 L bioreactor where Microcarrier A supported best growth with 38 fold increase at day 11, representing over 5 population doublings. This is consistent with microcarrier A yielding best day 7 fold increase in the small scale culture screen. However, the animal origin-free microcarriers B and C were not able to support bmMSCs in the bioreactor, yielding only 6 and 14 fold increases, respectively (data not shown).

**Table 1:** Expansion of aMSCs with various microcarriers in static or agitated small scale platforms.

	Animal origin-free	Day 7 fold increase		% Recovery		% Viability		Overall cell yield (E+04 cells / mL)		Rank
		*Petri*	*Spinner*	*Petri*	*Spinner*	*Petri*	*Spinner*	*Petri*	*Spinner*	

Microcarrier A^a^	No	24	24	35	52	98	95	4.5	10.3	3
Microcarrier B^a^	Yes	28	15	46	58	92	91	9.2	8.3	2
Microcarrier C^a^	Yes	21	16	59	86	98	93	11.6	18.8	1
Microcarrier D	Yes	28	12	25	84	97	95	2.9	13.5	4
Microcarrier E	Yes	29	16	27	46	96	97	3.3	5.3	5
Microcarrier F	No	24	17	22	30	91	90	1.9	2.4	6

^a^Microcarriers chosen for further evaluation in a bioreactor process.

**Figure 1:**
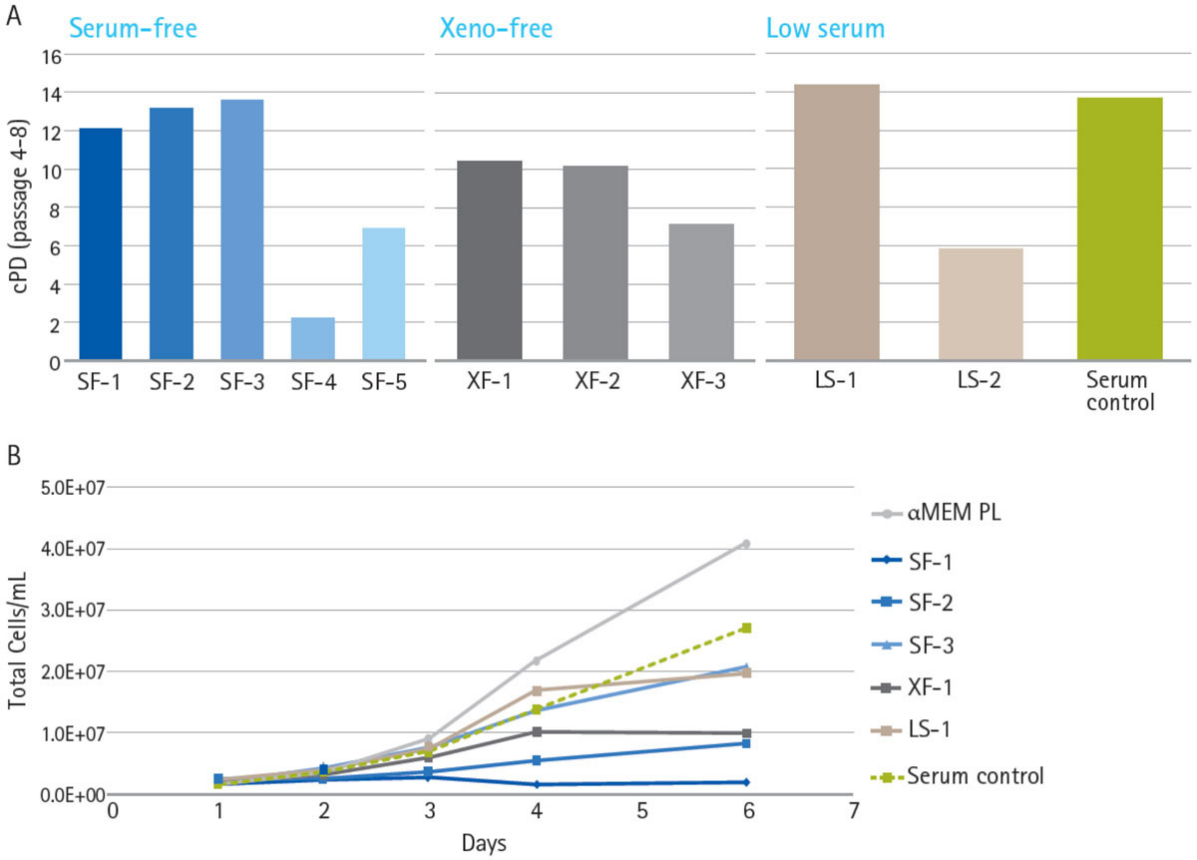
**Media that supported hMSC growth in planar culture exhibited variable performance in microcarrier-based suspension culture**. hMSCs were expanded in various serum-free, xeno-free or serum-containing media in either planar culture (a) or microcarrier-based culture (b, spinner flasks).

A number of serum-free (SF), xeno-free (XF) and low serum (LS) media were evaluated for expansion of bmMSCs in both planar and microcarrier-based suspension culture. Human platelet lysate (PL), a serum alternative, was also tested in the microcarrier-based culture. Wide variation in the support of growth was observed when bmMSCs were cultured for multiple passages in planar culture in different media (Figure 1A). Only 4 of the tested formulations, SF-1, SF-2, SF-3 and LS-1, had cumulative populations doubling similar to the DMEM FBS control. It should be noted that serum-free formulations may still contain animal-derived materials. These media, as well as XF-1 and αMEM PL were further evaluated in suspension culture (Figure 1B). Under agitated conditions, 2 of the 3 SF formulations did not support bmMSC growth, even though they performed well in planar culture. Robust growth was observed with αMEM PL, whereas SF-3 and LS-1 were within range of the serum control.

## Conclusions

The observation that some microcarriers and cell culture media that support robust MSC growth in static or planar culture do not perform in microcarrier-based suspension culture highlights the complex interaction between culture surface, media formulation and hydrodynamic forces introduced by agitation. Small scale platforms such as Petri dishes and spinner flasks are convenient tools for initial screening of many microcarriers or media, but a sufficient number of top performers need to be moved into bioreactor suspension culture for further evaluation. A better understanding of the underlying biological requirements of adherent-dependent cells in suspension culture will foster the development of high quality reagents that support expansion across platforms. The combination of serum-free systems with animal origin-free materials will be required to support the future implementation of large scale manufacturing solutions following clinical success of cell-based therapies.
